# Tips and tricks for addressing and responding to peer reviews

**DOI:** 10.1093/conphys/coag028

**Published:** 2026-05-07

**Authors:** Sean Tomlinson, Jeff C Clements, Steven J Cooke, Andrea Fuller, Bridget O’Boyle

**Affiliations:** School of Molecular and Life Sciences, Curtin University, Kent Street, Bentley, WA 6102, Australia; School of Biological Sciences, University of Adelaide, North Terrace, Adelaide, SA 5000, Australia; Fisheries and Oceans Canada, Gulf Fisheries Centre, 343 Université Avenue, Moncton, NB E1C 9B6, Canada; Department of Biological Sciences, University of New Brunswick, 100 Tucker Park Road, Saint John, NB E2L 4L5, Canada; Department of Biology, Institute of Environmental and Interdisciplinary Science, 1125 Colonel By Drive, Carleton University, Ottawa, ON K1S 5B6, Canada; Brain Function Research Group, Department of Physiology, Faculty of Health Sciences, University of the Witwatersrand, 7 York Rd, Parktown, 2193, South Africa; The Society for Experimental Biology, Lancaster University, Bailrigg, Lancaster LA1 4YW, UK

## Abstract

**Graphical Abstract:**
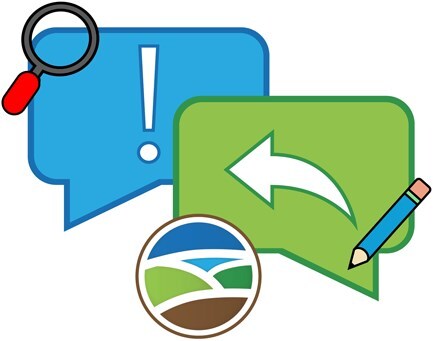

## Introduction

Although peer review is the fundamental process that strengthens the scientific method (Lock, 1985; Rowland, 2002), it is hindered by several well-recognized issues that undermine its objectivity and impartiality (Smith, 2006; Proctor *et al.,* 2023; Candal-Pedreira *et al.,* 2024). To help reduce potential conflicts or misunderstanding between reviewers and authors, members of the *Conservation Physiology* editorial team recently provided guidance on how reviewers can formulate comments that promote a more collaborative and collegial peer-review process (Clements *et al.,* 2025). Peer review is, however, a two-way process, and responding to it effectively not only increases the likelihood of publication success but also improves the quality of the resulting scientific reporting. Responding to peer review constructively is a challenging task for researchers at all career stages, from trainees to established researchers. Here, we present our perspectives and insights on how to most effectively respond to peer review, based on our experiences as editorial staff, editors and publishing researchers, as an extension of our recent advice on how to construct effective peer reviews (Clements *et al.,* 2025).

The limitations of the peer review process have been visited by numerous authors (Smith, 2006; Candal-Pedreira *et al.,* 2024) and generally focus upon reviewer misconduct and the responsibilities of reviewers and editors in safeguarding the integrity of the system (Tennant and Ross-Hellauer, 2020; Cooke *et al.,* 2024). In contrast, the roles and responsibilities of authors within the process receive far less attention, and are almost entirely unarticulated, aside from guidance dispersed through blogs and informal guidelines (e.g. guidelines from the British Ecological Society (https://www.britishecologicalsociety.org/wp-content/uploads/2024/12/BES-Peer-review-guide.pdf) and the Company of Biologists (https://journals.biologists.com/bio/pages/reviewer-guide)) and a handful of published works (e.g. Noble, 2017; Cushman, 2023; Hidouri *et al.,* 2024; Covvey *et al.,* 2025). The reasons for manuscripts failing to achieve publication have been broadly examined across numerous fields (e.g. McCance, 1995; Wu *et al.,* 2024), and typically focus on critical issues with experimental design, data presentation or analysis and appropriateness of inferences drawn (McCance, 1995). Rarely is a poor or inadequate response to peer reviewers' reports advocated as a reason for rejection. Nonetheless, there is importance in addressing this part of the peer review process because the response to peer review acts as the interlocutor to the reviewer in the conversation underpinning the review process. The community of scientists contributing to and engaging with scientific publishing is made stronger, more collegial and more productive if miscommunication and conflict in this interaction are minimized.

Despite widespread recognition of the value and need for formal training in peer review (Clements, 2020; Gerwing *et al.,* 2021; Feinmann, 2024), such training is not mandatory and remains rare (An *et al.,* 2023; Buser *et al.,* 2023; Willis *et al.,* 2023; Stupacher, 2025). The goal of this editorial is to further promote a collaborative and collegial peer review process at *Conservation Physiology*, and beyond. To help achieve this goal, we provide personalized direction on how to respond to reviewer comments constructively, and how to manage diverging opinions between authors and reviewers productively. Although this editorial is written from the perspective of *Conservation Physiology* editors, the guidance to authors, which we hope will particularly benefit early-career researchers, is broadly applicable across scientific journals and disciplines.

## Five tips for responding constructively to review

It is easy, in the position of a corresponding author, to feel as though the peer review process is a thinly veiled excuse to attack the work and the integrity of the research and the research team. Hopefully a well-considered and well-constructed review can mitigate this feeling. However, as a reviewer or editor, it can be equally destructive to the peer review relationship to have invested in providing a quality review only for it to be met with unconstructive, argumentative responses, or to be disregarded altogether. In this section, we briefly outline constructive approaches to receiving and responding to peer review comments and associated editorial decisions. In the text below, we provide five tips for crafting a constructive response to a reviewer comment. We consider the following reviewer comment and associated author response:

**Reviewer comment:** More sophisticated statistical analysis should be used as ANOVA is inadequate.

**Response**: The reviewer's comment is unjustified and lacks explanation; we disagree with the reviewer and have elected to keep the statistical analysis as is.

While author agreements with reviewer comments are, by nature, collegial, we specifically consider an example of a situation where authors disagree with and rebut a reviewer comment to show (below) that disagreements can still be communicated collegially.

### Tip 1: pick your battles

Peer review is a collaborative process, and differences of opinion will arise (see Tip 2). As authors, it is easy to become defensive over our work because of the substantial effort required to get a manuscript published. Some suggestions by reviewers can, consequentially, be seen as fatuous, semantic or overly pedantic. In such cases, author responses can sometimes take an overly defensive tone and even include *ad hominem* critiques of the reviewer. In the example above, part of the response is unnecessarily argumentative and hostile in responding to the reviewer’s suggestion, and can be toned back:

**Revised response**: We disagree with the reviewer and have elected to keep the statistical analysis as is.

[Note the removal of the first sentence in this Revised response].

While differing thoughts on a statistical approach may be worth refuting, reviewer suggestions are often easy to accommodate, and it is worth stepping back to ask whether the suggested change substantively compromises the meaning of the work, or whether accommodating the simple change is the best route forward. When a reviewer's suggestion is justified and can easily be addressed without escalating the disagreement, incorporating the change may provide the path of least resistance without altering the manuscript's message. This not only applies to minor suggestions but can also apply to more major suggestions, including statistical reanalysis as in our example above. Indeed, rather than rebutting, some authors may simply choose to employ a different statistical approach, as long as that statistical approach is valid, especially in the case where the altered analysis does not materially change the interpretation of the results. Ultimately, while authors can choose to refute or accept any reviewer comment, they should carefully consider whether reviewer suggestions are truly worth rebutting.

### Tip 2: refute, do not refuse

Disagreement is a part of scientific discourse, and even the most accommodating peer review is likely to uncover some points of contention. Reviewers can also be ‘wrong’, and a large proportion of peer reviews contain ‘incomplete, inaccurate or unsubstantiated critiques’ (Gerwing *et al.,* 2020). In circumstances where such incorrect reviews are provided, reviewers can miss critical points that are clearly stated in the initial manuscript, or criticize broadly accepted methods of which the reviewers may be unaware (Gerwing *et al.,* 2020). Disagreeing with a peer review is perfectly acceptable, but it is essential that any point of contention be clearly and objectively argued and refuted, rather than simply ignored. If reviewers' opinions have been sought, then the *de facto* implication is that their opinions are valuable. After investing effort in crafting a careful peer review report, a reviewer may find it dispiriting or frustrating when the suggestions have been dismissed without justification by authors, or else when authors respond primarily by contesting the perspective rather than addressing the substance of the feedback. In the case where this suggestion is to be refuted, the authors might write:

**Revised response:**  *Based*  *on the statistical design and the nature of our data, we feel that ANOVA is an appropriate statistical approach. As such*, we have elected to keep the statistical analysis as is.

An even better response would provide a sound explanation for the authors' logic, which can often be enough to dispel the issue completely:

**Revised response:**  *Our experimental design includes three experimental treatment groups: Control, Exp1 and Exp2. The sample size within each group is fully balanced (n = 30) and each experimental group was independently replicated three times. Assessments of residual normality and homogenous variances indicated that our data did not violate these assumptions of ANOVA*. Based on the statistical design and the nature of our data, we feel that ANOVA is an appropriate statistical approach. As such, we have elected to keep the statistical analysis as is.

### Tip 3: seek clarification

Despite what might be hoped, some reviewer feedback is just downright obscure. In viewing peer review as an academic discourse, it is legitimate to seek clarification on some points, either through contacting the handling editor or by seeking clarification in a response. In our example, the reviewer may have some other reason for suggesting an alternative statistical approach that the authors may have missed. A collegial response to the reviewer would leave open the possibility of altering the analysis if there is a logical and procedural reason for doing so. Therein, the response to the reviewer may read:

**Revised response:** Our experimental design includes three experimental treatment groups: Control, Exp1 and Exp2. The sample size within each group is fully balanced (*n* = 30) and each experimental group was independently replicated three times. Assessments of residual normality and homogenous variances indicated that our data did not violate these assumptions of ANOVA. Based on the statistical design and the nature of our data, we feel that ANOVA is an appropriate statistical approach. As such, we have elected to keep the statistical analysis as is. *Notwithstanding, we are not sure why the reviewer feels ANOVA is inadequate and will happily consider other statistical approaches if a more detailed rationale could be provided*.

### Tip 4: courtesy costs nothing

Receiving overly critical comments from the infamous ‘Reviewer 2’ is such a common experience in the peer review process that it has become a scientific meme (Peterson, 2020; Worsham *et al.,* 2022; Covvey *et al.,* 2025). In practice, the numerical label of this reviewer is irrelevant (Covvey *et al.,* 2025). What matters is the common perception and frequent experience of encountering a reviewer who is pedantic, strongly opinionated, or who simply misses the point of the manuscript they have been asked to evaluate. No matter how much effort a journal or its editorial board invests in guiding reviewers or fostering a constructive review culture, some inadequate or unconstructive reviews will slip through the cracks. At times, even good reviewers will have a bad day. And occasionally a reviewer may choose to remain involved after accepting an invitation, despite realizing upon reviewing the full manuscript that its scope extends beyond their expertise.

Whatever the circumstances that resulted in the perception that ‘Reviewer 2’ is obstructing the process, responding in kind is unproductive. At best, it might be worth asking the hard question of whether the manuscript is communicated as clearly as it could be. At worst, polite refutation is always the best policy. The reasons are twofold: firstly, if the manuscript is revised and goes back to the same reviewers, Reviewer 2 is not going to respond well to an aggressively worded rebuttal when they have taken time to review a manuscript (see Tips 1 and 2). Indeed, many authors start their response by expressing appreciation to the reviewers (even ‘Reviewer 2’) for taking the time to evaluate and comment on the manuscript, thereby establishing a collegial tone for the whole response letter. Secondly, the editors need to consider not just the well-being of the authors in the peer review process, but also that of the reviewers. A manuscript attached to an abusive response is likely to be rejected. Clearly, adopting a more conciliatory approach is likely to lead to a more favourable outcome:

**Revised response:**  *Thanks to the reviewer for this suggestion regarding statistics*. Our experimental design includes three experimental treatment groups: Control, Exp1 and Exp2. The sample size within each group is fully balanced (*n* = 30) and each experimental group was independently replicated three times. Assessments of residual normality and homogenous variances indicated that our data did not violate these assumptions of ANOVA. Based on the statistical design and the nature of our data, we feel that ANOVA is an appropriate statistical approach. As such, we have elected to keep the statistical analysis as is. Notwithstanding, we are not entirely sure why the reviewer feels ANOVA is inadequate and will happily consider other statistical approaches if a more detailed rationale could be provided. *We have nonetheless included written rationale for why we chose ANOVA in the text of the manuscript*.

### Tip 5: structure your responses

Reference to specific line numbers where changes have been made is a valuable aspect of your response, but even greater value is gained by quoting the specific changes made, and providing a tracked-changes version of the manuscript, so that these alterations can be easily identified (Cushman, 2023). For many journals, including *Conservation Physiology*, submission of a tracked-changes document is a required component of the revision process, but the value of that document is greatly enhanced when paired with a clear, well-structured document guiding reviewers and editors to the revisions. Given that the aim is to make it easy for reviewers and editors to identify the changes in the revised manuscript, we also advise that authors double-check that the line numbers referred to are correct prior to resubmitting the manuscript, and whether they refer to the tracked-changes or clean version of the manuscript. All too often, authors submit responses to reviewers and the line numbers are incorrect, resulting in confusion for editors and reviewers.

**Revised response:** Thanks to the reviewer for this suggestion regarding statistics. Our experimental design includes three experimental treatment groups: Control, Exp1 and Exp2. The sample size within each group is fully balanced (*n* = 30), and each experimental group was independently replicated three times. Assessments of residual normality and homogenous variances indicated that our data did not violate these assumptions of ANOVA. Based on the statistical design and the nature of our data, we feel that ANOVA is an appropriate statistical approach. As such, we have elected to keep the statistical analysis as is. Notwithstanding, we are not entirely sure why the reviewer feels ANOVA is inadequate and will happily consider other statistical approaches if a more detailed rationale could be provided. We have nonetheless included written rationale for why we chose ANOVA in the text *on Lines 100–105 of the clean version of the manuscript*:

*“Our experimental design included three experimental treatment groups: Control, Exp1 and Exp2. Since the sample size within each group was fully balanced (n = 30) and each experimental group was independently replicated three times. Since our data did not violate assumptions of residual normality and homoscedasticity, we analyzed the data using ANOVA.”*

## Additional tricks for crafting a constructive response

### Respond to all comments provided

In responding to peer review, it is important to remember that your revised manuscript will be assessed by the handling editor. It makes it easier and makes reviews quicker if every suggestion, comment and query is responded to clearly, even if the response is simply to accept the suggested change. The process moves more rapidly if reviewers and editors can clearly see that their advice has been carefully and thoughtfully received—even if some of the proposed changes are justifiably dismissed.

### Be considerate of languages other than English

Science is typically communicated in English, and English is not everybody’s first language (Cushman, 2023). When considering peer review as an author, be considerate of whether the reviewer has misunderstood you because of such a language barrier (either theirs or your own). Try to clarify their point and place your response in that context. Try to be consistent with terminology; lexical flair has its place, but not if it leads to confusion in reporting scientific advances. Most importantly, never assume a reviewer is inexpert because of a linguistic disconnect (Cushman, 2023). If a non-native English-speaking (or native English-speaking) reviewer is confused, that usually points towards opportunities to improve the clarity of your writing.

### Try to remain positive and open-minded

We have alluded here, and in our previous work (Clements *et al.,* 2025), to the common feelings of negativity and inadequacy that can result from critical peer review (Chrousos and Mentis, 2020; Covvey *et al.,* 2025). That is not the purpose of peer review, no matter how extensive or critical the feedback may be. Approaching peer review from the perspective that most peer reviewers are trying to improve the work (Covvey *et al.,* 2025) is important for preserving your well-being, as well as maintaining the integrity of the process. The emerging pattern, when tracing the revisions of our example response here, is one of increasing recognition of the reviewer's efforts, greater engagement with the underlying reason for disagreement, and an open-minded willingness to accept the reviewer's suggestion if the rationale is more clearly explained. Even though, in our most refined example here, the author continues to refute the reviewer's point, changes are made to the manuscript that clearly articulate the validity of the original statistical approach. An open-minded approach, implicitly assuming that the reviewer is not wrong, but probably has misunderstood the rationale as explained in the original draft, has ultimately improved the communicative power of the manuscript and maintained a positive conversation between the author and the reviewer.

### Sleep on it

Advice on how to respond to peer review often recommends drafting your response in two phases, with the first draft revised or even discarded entirely (Noble, 2017). This initial draft serves as a space to vent frustration with any aspects of the feedback that seem unfair, and to deal with the disappointment of not having the manuscript accepted for publication. Taking some time to process this disappointment, and to allow the frustration to subside before approaching the response to your peer reviewers with as open a mindset as possible is advised (Covvey *et al.,* 2025). We agree with this advice and generally find that the most productive responses to peer review are written after a night of sleep, and consultation with the other authors of the manuscript.

### Submit your revision on time, or request an extension from the editorial office

Typically, all decisions on manuscripts will contain a date by which the editor would like you to return your revised manuscript. This deadline allows publishers and editorial teams to maintain efficient workflows and reduce times to publication. Shorter turn-around times are also more efficient for editors and reviewers as there is less need for refamiliarization with the manuscript contents. That said, sometimes the feedback requires some substantial reworking, or even a new line of inquiry that would substantially strengthen the work, and revisions may take longer than expected. Being unable to meet a resubmission deadline is certainly understandable under these scenarios. If, at the time you receive a decision, you anticipate that the required revisions will be substantial, you may respond to the decision letter and ask for an extended timeframe. Should the scope of work expand during the revision process, or your time be taken up by unexpected obligations, simply notify the editorial office that you require extra time. It is unlikely that you will be refused the opportunity to resubmit your work later, but proactive communication ensures that all parties remain informed.

### Contact the editor if things do not go well

The standards to which reviewers are expected to adhere by most journals, including *Conservation Physiology,* are publicly available and transparent to both reviewers and authors alike, and emphasize the need for reviewers to be rigorous but considerate. Nevertheless, despite the best efforts of the journal and its editorial team, suboptimal reviews do occur. Constructive feedback from authors helps the editorial team identify reviews that fall well outside the standards of the journal and recognize reviewers who repeatedly fail to meet expectations, especially reviews that are overly opinionated, contain discriminatory language or suggest unreasonable demands or *quid pro quo* arrangements. Notifying editors of such situations can reduce the likelihood of similar issues arising in the future. Such notification can be done by correspondence with the editor through the journal's submission system, or by including feedback in the cover letter of the revision. It is important to keep in mind that all aspects of constructive review discourse mentioned above still apply—editorial contact is not an opportunity to express frustration in an unconstructive or unprofessional manner. However, considering that research is undertaken by a community of peers, and that peer review is a self-correcting process to improve science, there is value in treating feedback as a two-way process when reviewers egregiously fail to uphold their responsibilities.

### What should you do if you believe your manuscript was rejected unjustly?

When an editor rejects a paper without clear justification or with reasoning the authors believe is flawed, the most constructive step is to request clarification and succinctly point out the specific issues you believe were not fairly evaluated. A concise, evidence-based appeal to the editor can prompt reconsideration, ensuring that your manuscript receives proper scrutiny.

## Closing remarks

In this editorial, we provide authors with practical guidance for responding to peer review in ways that may help to smooth the dialogue between authors, reviewers and editors. Many of these suggestions align with existing but newly emerging discussions (Cushman, 2023; Covvey *et al.,* 2025), suggesting that the role of authors in the peer review process has been under-recognized historically. While training in the peer review process is viewed as essential, it remains uncommon in science (An *et al.,* 2023; Buser *et al.,* 2023; Willis *et al.,* 2023; Stupacher, 2025). Guidance on how to respond to peer reviewers, especially for early-career scientists, appears to be even more *ad hoc*. Responding effectively to peer review not only streamlines the publication process but also helps safeguard the well-being of all participants in the process, including reviewers, editors and authors. We hope that this editorial is helpful for authors resubmitting their work to *Conservation Physiology*, and to other journals, promoting constructive engagement and strengthening the collective scientific endeavour.
